# Potential of soil amendments (Biochar and Gypsum) in increasing water use efficiency of *Abelmoschus* esculentus L. Moench

**DOI:** 10.3389/fpls.2015.00733

**Published:** 2015-09-11

**Authors:** Aniqa Batool, Samia Taj, Audil Rashid, Azeem Khalid, Samia Qadeer, Aansa R. Saleem, Muhammad A. Ghufran

**Affiliations:** ^1^Department of Environmental Sciences, Pir Mehr Ali Shah Arid Agriculture University RawalpindiRawalpindi, Pakistan; ^2^Department of Environmental Sciences, International Islamic University, IslamabadPakistan; ^3^Key Laboratory of Mountain Ecological Restoration and Bioresource Utilization & Biodiversity Conservation Key Laboratory of Sichuan Province, Chengdu Institute of Biology, Chinese Academy of Sciences, ChengduChina

**Keywords:** *Abelmoschus*, biochar, water use efficiency, photosynthesis, stomatal conductance, transpiration rate

## Abstract

Water being an essential component for plant growth and development, its scarcity poses serious threat to crops around the world. Climate changes and global warming are increasing the temperature of earth hence becoming an ultimate cause of water scarcity. It is need of the day to use potential soil amendments that could increase the plants’ resistance under such situations. Biochar and gypsum were used in the present study to improve the water use efficiency (WUE) and growth of *Abelmoschus esculentus* L. Moench (Lady’s Finger). A 6 weeks experiment was conducted under greenhouse conditions. Stress treatments were applied after 30 days of sowing. Plant height, leaf area, photosynthesis, transpiration rate (Tr), stomatal conductance and WUE were determined weekly under stressed [60% field capacity (F.C.)] and non-stressed (100% F.C.) conditions. Stomatal conductance and Tr decreased and reached near to zero in stressed plants. Stressed plants also showed resistance to water stress upto 5 weeks and gradually perished at sixth week. On the other hand, WUE improved in stressed plants containing biochar and gypsum as compared to untreated plants. Biochar alone is a better strategy to promote plant growth and WUE specifically of *A. esculentus*, compared to its application in combination with gypsum.

## Introduction

Drought is a long period of weather dryness due to lack of rainfall in any area that creates a serious imbalance of water and moisture deficiency for agriculture. Climate change and global warming are two basic environmental issues that are caused by anthropogenic activities making agricultural crisis even more severe. High temperature and severe weather conditions are the causes of flash floods, drought and glacier melting. Due to these climatic changes, earth is becoming warmer and drier and ultimately resulting in water scarcity.

Water stress has become a worldwide problem, as it is a threat to the sustainability of agriculture (Shao et al., 2005). Asian countries including Pakistan are agriculture based countries and their agricultural productivity and yield is adversely affected due to water shortage. This water crisis affects the productivity as well as yield of crops (Huaqi et al., 2002). In arid and semiarid regions, water stress is also faced by plants in two conditions, either when water supply is reduced or the rate of transpiration may become very high than the normal rate (Tan and Weaver, 1997).

In contrast, some plant species are resistant to drought stress. Such tolerance in plants may vary from specie to specie due to the adaptation of different mechanisms (Chaitanya et al., 2003). Therefore, there are some crops that can grow under deficit water by adopting different drought tolerance mechanisms such as acclimation (Bohnert and Sheveleva, 1998), osmotic adjustment (Saab et al., 1992), hormonal control and stomatal closure etc. (Sanchez-Blanco et al., 2002). In arid and semiarid regions water use efficiency (WUE) of plants indicates the crop productivity as well as water use by different plants (Yu et al., 2004). It is measured with reference to water and CO_2_ used during the process of photosynthesis (Swarthout et al., 1989).

There are some soil amendments that increase drought resistance for plants and also improve the WUE. Biochar is an example of such amendments which are used as a solution of drought stress for many plants. It contains 80% of carbon that has the ability to conserve water and nutrients for plants and hence considered as drought enduring material (Novak et al., 2009; Major et al., 2010). The application of biochar to soil is useful in many respects. It improves the soil microbial interaction and activity (Pietikainen et al., 2000), shows retention of nutrients in the soil (Wardle et al., 1998; Lehmann et al., 2003) and at the same time increases water holding capacity of the soil (Laird et al., 2010). Biochar is applied along with any fertilizer; thus the crop shows enhanced productivity toward such amendments (Steiner et al., 2007).

Different plant species can be used to prepare biochar from their biomass. In many subtropical areas of the world *Lantana camara* is an invasive plant that not only affects the grazing lands but also reduces the soil nutrients for other useful plants. To overcome this problem, *L. camara* can be removed from such lands and could be used to prepare biochar from its biomass solving the disposal issue.

Many studies have investigated the impact of biochar addition on the performance and drought resistance of plants. As soil amendments like biochar has been proposed as a potential measure to increase agricultural productivity, by improving water holding capacity, along with enhanced nutrient retention of the soil. Apart from biochar, gypsum is also a well known soil amendment to enhance the quality of soil as well as its water retention capacity. Gypsum is known to improve the plant productivity (Scott et al., 1993), enhances nutrient availability to plants (Vyshpolsky et al., 2010), improves soil moisture content by altering water holding capacity (Al-Oudat et al., 1998), improves soil structure, infiltration rate and enhances water movements in soil (Chi et al., 2012) and also promotes the root system of plants (Ritchey et al., 1995). As biochar and gypsum are porous materials these possess high water holding capacity. Hence, in the present study, the beneficial effect of biochar and gypsum are supposed to be further enhanced by combined application of biochar and gypsum on *Abelmoschus esculentus* L. Moench (commonly called as “Okra”). For the present study Lady’s finger was selected, as it requires short duration to complete its life cycle. In addition, it is a common crop which has an important nutritious value as containing carbohydrates, proteins, iron, Vitamin A, B, and C (Dilruba et al., 2009).

The current study was aimed:

• To improve the WUE of *A. esculentus* L. Moench with application of biochar and gypsum as soil amendments
• To assess the impact of biochar and gypsum on different plant parameters (leaf area, plant height, transpiration, photosynthesis, stomatal conductance)

## Materials and Methods

### Soil Analysis

Soil was air-dried, ground and sieved through 2 mm sieve. It was analyzed for different parameters. Soil pH and electrical conductivity (EC) was measured by using modified glass electrode method (Page, 1982). Moisture content was determined by gravimetric method (Hesse, 1971). Soil texture was also assessed using modified Bouyoucos Hydrometric method (Sheldrick and Wang, 1993). Furthermore, organic matter was calculated by Walkley-Black method (Nelson and Sommers, 1982).

### Biochar Preparation and Analysis

Plant biomass of *L. camara* was dried and chopped into small pieces. Biochar was prepared by pyrolysis of stems of *Lantana* at 450°C using reactor according to procedure described by Odesola and Owoseni (2010). Biochar was also characterized for different parameters. Biochar pH, EC and ash content were determined according to procedure described by Ellen et al. (2010). Moreover, moisture content was also calculated by procedure given in ASTM (2007) 1762-84. Furthermore, bulk density was measured using modified procedure described by Ozcimen and Karaosmanoglu (2004).

### Experimental Design

The pot experiment was conducted by sowing the seeds of Lady’s finger (*A. esculentus* L. Moench) before the application of the soil amendments. The pots of ∼5 kg capacity (17.7 cm high × 21.2 cm diameter) were used for the experiment. In each pot 4 kg soil with the mixture of soil (ground, air-dried and sieved) and sand in 3:1 ratio was taken (Kholová et al., 2010). The experiment was Randomized Complete Block Design (RCBD). There were six treatments with biochar and gypsum and two treatments of water stress with three replications for the assessment of WUE. Therefore, the experimental units were 36 which were used to conduct the proposed study. Treatments were applied as follows in **Table 1**.

**Table 1:** Details of soil amendments (biochar and gyupsum) applied at different water levels.

Treatments	Water level	Biochar	Gypsum
	(%)	(%)	(%)
T_1_	100	0	0
T_2_	60	0	0
T_3_	100	1 (10 g/kg soil)	0
T_4_	60	1 (10 g/kg soil)	0
T_5_	100	3 (30 g/kg soil)	0
T_6_	60	3 (30 g/kg soil)	0
T_7_	100	1 (10 g/kg soil)	0.1 (1 g/kg soil)
T_8_	60	1 (10 g/kg soil)	0.1 (1 g/kg soil)
T_9_	100	3 (30 g/kg soil)	0.1 (1 g/kg soil)
T_10_	60	3 (30 g/kg soil)	0.1 (1 g/kg soil)
T_11_	100	0	0.1 (1 g/kg soil)
T_12_	60	0	0.1 (1 g/kg soil)

### Application of Treatments

Five Seeds of lady’s finger (*A. esculentus* L. Moench) were sown in each pot planted in biochar and gypsum amended soil. The rate of biochar application was based on the previous study conducted by Ellen et al. (2010). After the germination of seeds, the seedlings were thinned upto two in each pot. Thirty days after sowing, pots were maintained at 100 and 60% field capacity (F.C.). Water treatments were imposed according to Sankar et al. (2008). Pots were covered with polythene bags to avoid the moisture loss through soil evaporation (Sankar et al., 2008). At the same time, pots were weighed daily on digital field balance and moisture was maintained upto the field capacity (100, 60%) every day for 3 weeks. After 3 weeks, the pots of 60% F.C. were given the stress and continuously 60% of daily water loss was returned to all stressed plants until the termination of the experiment. The stress was given to the plants to evaluate the resistance of Lady’s finger by applying biochar and gypsum. The experiment was continued upto 6 weeks until the stomatal conductance of plants reached near to zero. The total duration of stress was 6 weeks (i.e., 45 days) and the measurements for the growth parameters were taken on 15th and 30th day.

### Plant Growth Parameters

#### Plant Height

The individual plant heights were measured at 15th and 30th day after stress (DAS) induction. Each time the total height of every plant was measured from the base to the apex of the stem using measuring tape (in centimeter).

#### Leaf Area

Leaf area was also measured at 15th and 30th DAS induction according to procedure described by Masinde et al. (2006).

#### Physiological Parameters

Stomatal conductance, transpiration rate (Tr) and net photosynthesis were determined weekly using CIRAS 2 Portable Photosynthetic System (IRGA). A fresh and fully grown leaf was selected and enclosed in the cuvette of infrared gas analyzer (IRGA) to determine the values (Fang et al., 2012).

#### Water Use Efficiency

At leaf level WUE is the ratio of photosynthesis and Tr. It was determined by the given formula:

WUE = Pn/Tr

Where WUE is the WUE of plants, Pn is net photosynthesis rate and Tr is the transpiration rate (Jianlin et al., 2008).

#### Biomass

At the termination of experiment (week six), plant fresh weight was measured using digital balance. After harvesting all the plants were dried at 70°C for 72 h and again dry weights were measured. The difference in weight was calculated as the biomass of plants.

#### Statistical Analysis

Through MS Excel 2007 two way analysis of variance (ANOVA) was used to analyze the data statistically at 5% level of significance.

## Results

### Soil Analysis

Before and after the application of treatments, the soil was analyzed for the physical and chemical parameters such as pH, EC, moisture content, soil texture and organic matter. These were determined for all the treatments including biochar and gypsum as well. Results of these parameters are presented in **Table 2**.

**Table 2:** Physico-chemical Characteristics of Soil after the application of treatments.

Treatments	pH	EC	Moisture	Organic Matter	Soil texture
		(μS/cm)	(%)	(%)	
Control	7.28 ± 0.01	3.03 ± 0.005	1.21 ± 0.1	0.5 ± 0.03	Sandy loam
1% B	9.06 ± 0.01	13.01 ± 0.01	14 ± 0.02	1.7 ± 0.07	Sandy loam
3% B	8.81 ± 0.01	9.94 ± 0.01	18 ± 0.01	1.9 ± 0.01	Sandy loam
1% B + G	7.71 ± 0.005	6.65 ± 0.01	25 ± 0.03	3.4 ± 0.06	Sandy loam
3% B + G	7.81 ± 0.01	5.56 ± 0.01	33 ± 0.07	2.9 ± 0.01	Sandy loam
0.1% G	6.56 ± 0.01	8.17 ± 0.01	14 ± 0.06	3.4 ± 0.01	Sandy loam

*B, Biochar, G, Gypsum.*

Before the start of experiment, pH of soil was 7.28 and categorized as neutral soil. The EC of soil was 3.03 μS/cm. The moisture content present in air-dried soil was 1.21 percent. The percentage of sand, silt and clay was 60, 15, 25 percent, respectively. Therefore, soil textural class was sandy loam. Organic matter was low in soil before the application of soil amendments. It was 0.47% in soil without any amendment.

As the soil textural class was sandy loam, it has been observed that biochar and gypsum gave the positive response toward these soil parameters. Biochar pH, EC, moisture content and organic matter has been increased in all treated plants as compared to control soil.

### Biochar Analysis

Biochar was also analyzed for different parameters like pH, EC, moisture content, ash content and bulk density. Results of these parameters are presented in **Table 3**.

**Table 3:** Physical characteristics of biochar.

Biochar parameters	Values
pH	9.52 ± 0.01
EC (μS/cm)	23.7 ± 0.1
Moisture content (%)	4.03 ± 0.01
Ash content (%)	23.98 ± 0.01
Bulk density	0.5 ± 0.02

### Plant Height

**FIGURE 1:**
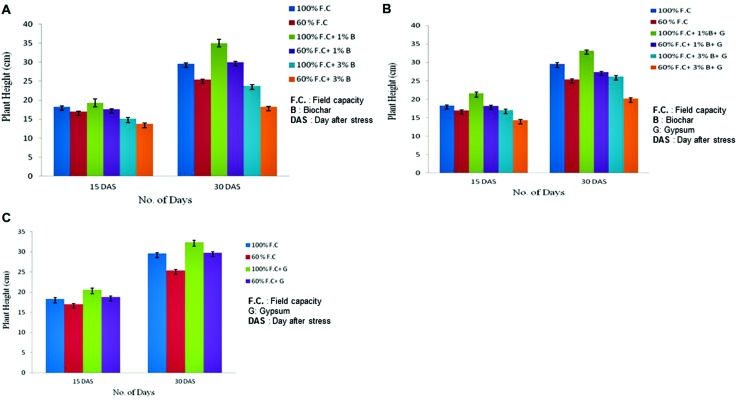
**(A)** Comparison of plant height between control and biochar treatments under stressed [60% field capacity (F.C.)] and non-stressed (100% F.C.) conditions. **(B)** Comparison of plant height between control and combination of biochar and gypsum treatment under stressed and non-stressed conditions. **(C)** Changes in plant height of control and gypsum treated plants under stressed and non-stressed conditions.

Plant height measured at 15th and 30th DAS showed highly significant difference (*P* ≤ 0.01) in biochar treated plants as compared to control (**Figure 1A**). Plant height was higher in non-stressed (100% F.C.) plants as compared to the stressed (60% F.C.) ones. Among all these treatments (100% F.C. + 0% B, 100% F.C. + 1% B, 100% F.C. + 3% B) the plant height was higher in 1% biochar treated plants as compared to control and 3% biochar. It was 35 cm as compared to 0 and 3% where the heights were 29.6 and 23.8 cm respectively.

Similarly, at 60% F.C. of the same treatments, plant height was higher with 1% Biochar as compared to 0 and 3% Biochar. Moreover, non-stressed plants (100% F.C.) were also compared with stressed plants (60% F.C.) and also found significantly different (*P* ≤ 0.01) in height. Plant height was privileged in non-stressed plants as compared to stressed plants. Therefore, 1% biochar application rate was found to be more effective than other rates.

Comparison of plant height between control and combination of biochar and gypsum treatments is shown in **Figure 1B**. The plant height was elevated in biochar and gypsum treated plants as compared to control where no soil amendments were applied. The highly significant results were found for the plant height of all treatments at *P* ≤ 0.01. Among all these treatments (100% F.C + 0% biochar + 0% gypsum, 100% F.C. + 1% biochar + 0.1% gypsum, 100% F.C + 3% biochar + 0.1% gypsum) the plant height in all treatments increased throughout the experiment. But it was high in plants with 1% biochar + 0.1% gypsum (33.2 cm) as compared to other treatments. Similar trend was found in stressed plants (60% F.C.) and 1% biochar + gypsum was proved more beneficial soil amendment.

Gypsum also has significant effect on the growth parameters of plants if treated alone. Comparison of plant height between control and gypsum is presented in **Figure 1C**. Gypsum has also improved plant height as compared to control where no amendment was added as soil supplement. The highly significant results were obtained for plant height in all treatments (*P* ≤ 0.01). Among all these treatments (100% F.C. + 0% G, 60% F. C. + 0% G, 100% F.C. + 0.1% G and 60% F.C. + 0.1% G), maximum plant height was observed in gypsum applied plants (32.4 cm) as compared to control (29.6 cm). On the other hand plant height was maximum in non-stressed plants as compared to stressed.

### Leaf Area

**FIGURE 2:**
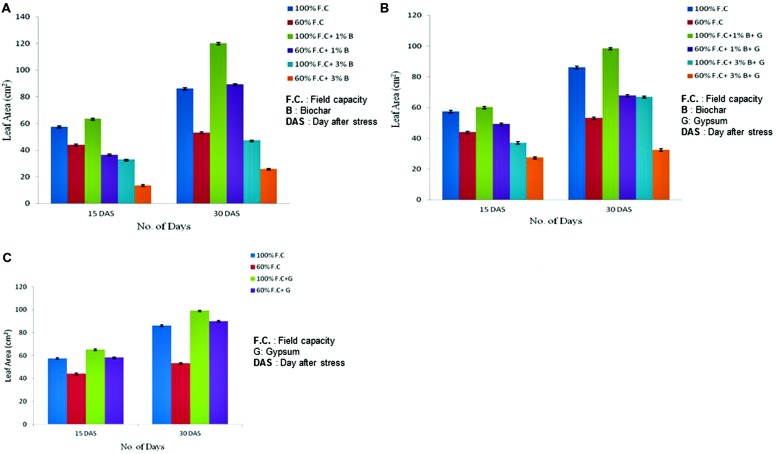
**(A)** Changes in leaf area of control and biochar applied plants under stressed (60% F.C.) and non-stressed (100% F.C.) conditions. **(B)** Comparison of leaf area between control and combination of biochar and gypsum treated plants under stressed and non-stressed conditions. **(C)** Comparison of leaf area of control and gypsum treated plants under stressed and non-stressed conditions.

Leaf area was also measured at 15th and 30th day of stress (DAS) for all stressed and non-stressed plants. Comparison of leaf area between control and biochar treatments is graphically presented in **Figure 2A**. The highly significant results (*P* ≤ 0.01) were found for all treatments. An increasing trend was observed among all these treatments (100% F.C., 100% F.C. + 1% B, 100% F.C. + 3% B). But in T2 100, maximum leaf area was observed (120.2 cm^2^) as compared to T1 100 and T3 100 where it was just 86.24 and 47.36 cm^2^, respectively. In the same way, in stressed treatments (60% F.C., 60% F.C. + 1% B, 60% F.C. + 3% B) same trend was found and leaf area was also high with 1% B at 60% F.C. In contrast, leaf area was larger in non-stressed plants (100% F.C.) than stressed one (60% F.C.).

Effect of combination of biochar and gypsum was also observed. The highly significant results (*P* ≤ 0.01) were also found when leaf area of control plants was compared with the combination of biochar and gypsum treated plants. Comparison of treated and untreated plants is graphically represented in **Figure 2B**. Among all treatments (100% F.C., 100% F.C. + 1% B + 0.1% G, 100% F.C. + 3% B + 0.1% G), leaf area was elevated in (1% B + 0.1% G) at 100% F.C. as compared to other treatments (0% B + 0% G and 3% B + 0.1% G). Therefore, at 100% F.C., 1% B + 0.1% G was considered to be more effective to increase the growth parameter as compared to other treatments. In addition, the same rates of biochar at 60% F.C. also showed the same trend.

Apart from biochar, gypsum alone is also effective for plants as obvious from the comparison of leaf area between control and gypsum (**Figure 2C**). The highly significant results (*P* ≤ 0.01) were found among all treatments (100% F.C. + 0% G, 100% F.C. + 0.1% G, 60% F.C. + 0% G and 60% F.C. + 0.1% G) showing maximum leaf area (90.12 cm^2)^ in gypsum treated plant as compared to control (86.24 cm^2^) with no gypsum application. At 60% F.C. similar trend was observed. Water has significant effect on the growth parameters of plants.

### Stomatal Conductance

**FIGURE 3:**
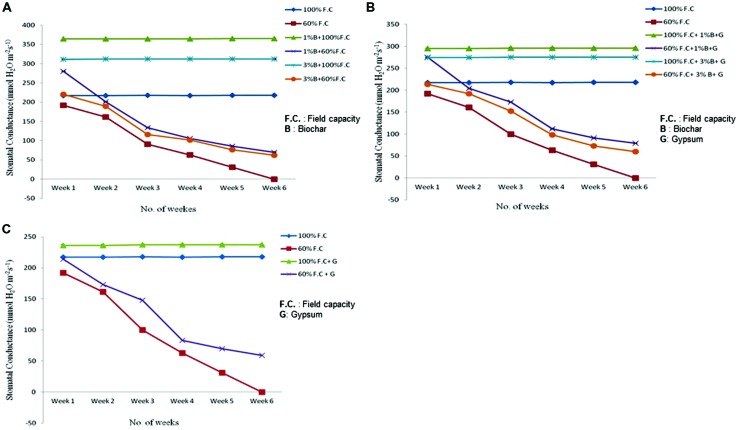
**(A)** Comparison of stomatal conductance of control and biochar amended plants under stressed (60% F.C.) and non-stressed (100% F.C.) conditions. **(B)** Comparison of stomatal conductance of control and combination of biochar and gypsum treated plants under stressed and non-stressed conditions. **(C)** Comparison of stomatal conductance of control and gypsum applied plants under stressed and non-stressed conditions.

At the start of experiment stomatal conductance was measured of all treated and untreated plants. Comparison of stomatal conductance between control and biochar treatments is shown in **Figure 3A** depicting highly significant difference (*P* ≤ 0.01) among all treatments. In all non-stressed plants (100% F.C. + 0% B, 100% F.C. + 1% B, 100% F.C. + 3% B) the stomatal conductance remained constant from week 1 to week 6 as these were maintained at 100% F.C. daily. The stomatal conductance was very high (364 mmolH_2_O m^-2^s^-1^) in 1% biochar as compared to 0 and 3% where it was 217 and 311 mmolH_2_O m^-2^s^-1^ respectively. On the other hand, in stressed plants (60% F.C. + 0% B, 60% F.C. + 1% B, 60% F.C. + 3% B) the conductance decreased very slowly up to the third week, until their F.C. was maintained at 60%. After 3 weeks when stress was increased, the sudden decline was observed in their stomatal conductance. Biochar showed stability at the intense stress and stomatal conductance was decreased upto 70 and 62 mmolH_2_O m^-2^s^-1^ in 1 and 3% B, respectively as compared to 0% B where it reached near to zero at the 6 week of experiment.

Besides biochar alone, the combination of biochar with gypsum has also shown significant results (*P* ≤ 0.01). The stomatal conductance of control and soil amended plants is graphically presented in **Figure 3B**. Like biochar treatment, similar results were found for the combination of biochar and gypsum. At 100% F.C. of all treatments (0% B + 0% G, 1% B + 0.1 % G, 3% B + 0.1% G) stomatal conductance remained the same throughout the experimental period. On the other hand in stressed treatments i.e., at 60% F.C. for all treatments (0% B + 0% G, 1% B + 0.1% G, 3% B + 0.1% G) it was decreased upto zero for 0% B + 0% G as compared to others where it didn’t reach to zero at the end of the experiment. Therefore, biochar and gypsum gave the resistance to plants under drought stressed conditions.

Gypsum alone also had positive effect to improve the stomatal conductance of plants. Stomatal conductance of control and gypsum applied plants is presented in **Figure 3C**. In all treatments (100% F.C. + 0% G, 100% F.C. + 0.1% G), no change was observed in stomatal conductance of plants. In addition, stomatal conductance was higher in gypsum treated plants (236 mmolH_2_O m^-2^s^-1^) as compared to control (217mmol H_2_O m^-2^s^-1^). In contrast, at 60% F.C. (60% F.C. + 0% G, 60% F.C. + 0.1% G) stomatal conductance was gradually decreased upto 3 weeks then it was decreased rapidly upto zero in 60% F.C. with no gypsum as compared to gypsum treated plants where it was 59 mmolH_2_O m^-2^s^-1^. Hence, gypsum and biochar have increased the soil moisture and ultimately their resistance and plants got maximum value of stomatal conductance.

### Transpiration

**FIGURE 4:**
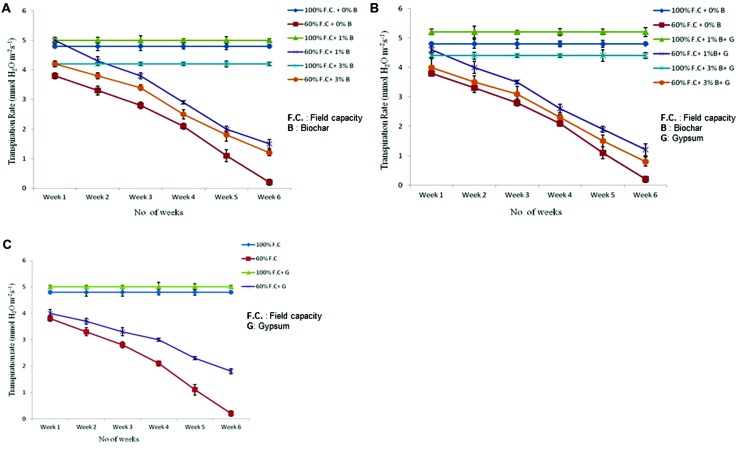
**(A)** Comparison of transpiration rate of control and biochar treated plants under stressed (60% F.C.) and non-stressed (100% F.C.) conditions. **(B)** Comparison of transpiration rate of control and combination of biochar and gypsum applied plants under stressed and non-stressed conditions. **(C)** Transpiration rate of control and gypsum treated plants under stressed and non-stressed conditions.

Transpiration rate of control and biochar treated plants was also compared presented graphically in **Figure 4A**. The results were highly significant (*P* ≤ 0.05) when Tr was compared between control and biochar treatments. Among all non-stressed treatment plants (100% F.C. + 0% B, 100% F.C. + 1% B, 100% F.C. + 3% B), the Tr was not significantly changed throughout the experiment as their F.C. was maintained at 100%, everyday. On the other hand, in stressed plants (60% F.C. + 0% B, 60% F.C. + 1% B, 60% F.C. + 3% B) the Tr was reduced slowly upto 3 weeks when their F.C. was maintained at 60%. After 3 weeks, plants were given stress and 60% of water loss was returned to all plants. At this stage, a rapid decline was observed in the rate of transpiration. Finally, it was reached near to zero at the end of the experiment in 0% biochar.

The combination of biochar with gypsum has also significant impact on the rate of transpiration. The combination of biochar and gypsum has also significant effect on the Tr of plants. Comparison of Tr between control and biochar and gypsum amended soil is presented in **Figure 4B**. Among all these treatments, (100% F.C. + 0% B + 0% G, 100% F.C. + 1% B + 0.1% G, 100% F.C. + 3% B + 0.1% G) no change was observed throughout the experiment. On the other hand, at 60% F.C. (0% B + 0% G, 1% B + 0.1% G, 3% B + 0.1% G) the Tr was gradually reduced upto 3 weeks and then suddenly decreased and reached near to zero at the end of experiment.

Besides the combination with biochar, gypsum alone has also significant effect on the Tr. Tr was compared between control and gypsum and graphically presented in **Figure 4C**. Like other treatments, same trend was observed with gypsum treatments at 100% F.C as well as 60% F.C. At 100% F.C. of all treatments (0% G and 0.1% G) Tr remained the same because plants were daily watered at 100% F.C. On the other hand, in case of 60% F.C. (0% G and 0.1% G) it was gradually decreased and reached near to zero at the end of the experiment.

At 100% F.C. the Tr was not altered because water loss was returned daily to the plants. Therefore it remained constant throughout the experiment. On the other hand at 60% F.C, only 60% of daily water loss was returned, so with the passage of time plants became sensitive and reached near to dry condition.

### Photosynthesis

**FIGURE 5:**
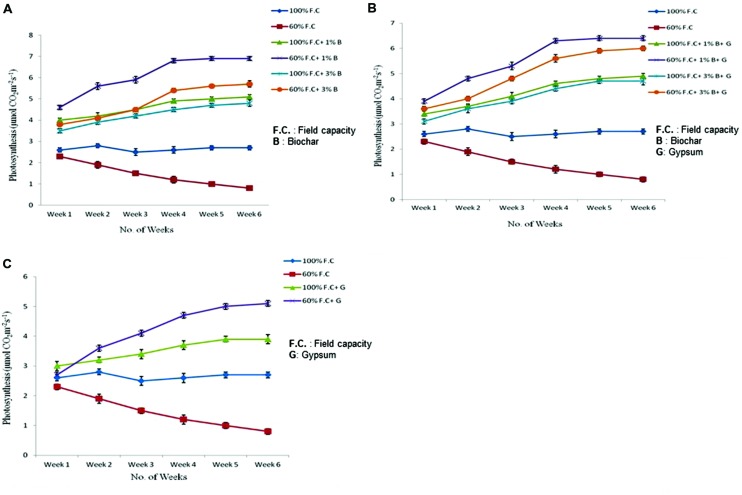
**(A)** Comparison of photosynthetic rate of control and biochar applied plants under stressed (60% F.C.) and non-stressed (100% F.C.) conditions. **(B)** Comparison of photosynthetic rate of control and combination of biochar and gypsum treated plants under stressed and non-stressed conditions. **(C)** Comparison of photosynthetic rate of control and gypsum treated plants under stressed and non-stressed conditions.

Photosynthesis of all plants was also measured throughout the experiment. The highly significant results (*P* ≤ 0.01) were found for all treatments (**Figure 5A**). At 100% F.C. of all treatments (**T1 100** = 100% F.C. + 0% B; **T2 100** = 100% F.C. + 1% B; **T3 100** = 100% F.C. + 3% B), a slight change was observed in the photosynthesis rate (Pn) of plants. Pn was higher in T2 100 (5.1 μmolCO_2_m^-2^s^-1^) as compared to T1 100 and T3 100 (2.7 and 4.8 μmolCO_2_m^-2^s^-1^), respectively. On the other hand an increasing trend was observed at 60% F.C. along with biochar (1 and 3% B) as compared to control (0% B) where it was reduced at the end of the experiment. Pn was higher in 1% B at 60% F.C. and increased from 4.6 to 6.9 μmol CO_2_m^-2^s^-1^ from week one to week six. Among all these treatments 1% B + 60% F.C. has shown significant impact on the Pn under drought stressed condition and proved effective treatment as compared to other treatments.

Comparison of photosynthesis between control and combination of biochar and gypsum treatments is presented in **Figure 5B**. The results were also found significant (*P* ≤ 0.01) among all the treatments. At 100% F.C. higher Pn was observed in combination of biochar and gypsum treatment as compared to control where no amendment was applied. Among all these treatments, (0% B, 1% B, and 3% B) 1% B was more effective at 100% F.C. than others. In contrast, Pn gradually increased in 60% F.C. in biochar treatments (60% F.C. + 1% B, 60% F.C. + 3% B) than control (60% F.C. + 0% B). In these plants, Pn was elevated from 3.9 to 6.4 μmol CO_2_m^-2^s^-1^. Similarly, 1% biochar along gypsum has also shown positive impacts than other treatments.

Photosynthesis rate of control and gypsum amended plants has also been compared (graphically presented in **Figure 5C**. At 100% F.C. (0% G and 0.1% G) like other treatments a slight change was observed in the Pn throughout the experiment. But gypsum treated plants gave high rate of photosynthesis (3.9 μmol CO_2_m^-2^s^-1^) as compared to control (2.7 μmol CO_2_m^-2^s^-1^). On the other hand, at 60% F.C. (0% G and 0.1% G) the photosynthesis was higher because gypsum prevents the water loss and ultimately increases the moisture and Pn. In gypsum applied plants, it was increased from 2.7 to 5.1 μmol CO_2_m^-2^s^-1^as compared to control where it was reduced from 2.3 to 0.8 μmolCO_2_m^-2^s^-1^.

Gypsum is considered more effective for plants growth as compared to other fertilizers under water stressed conditions. In all the treatments the trend was same in stressed and non-stressed plants. But positive results were obtained in biochar and gypsum amended treatments than control. In present study, net photosynthesis was improved.

### Water Use Efficiency

**FIGURE 6:**
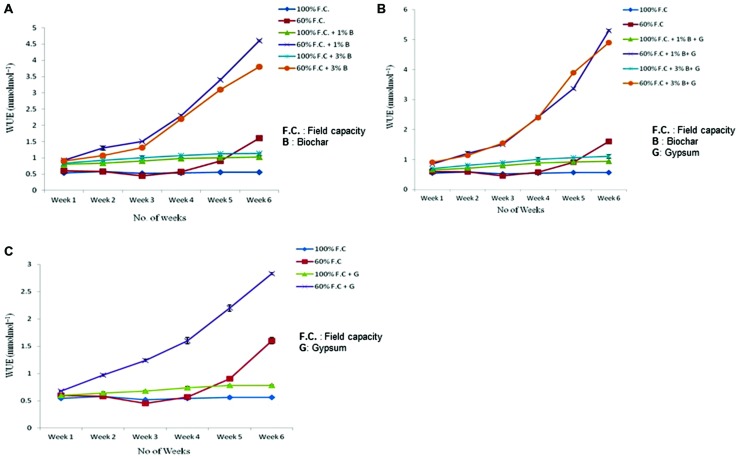
**(A)** Effect of biochar treatments on water use efficiency of stressed and non-stressed plants. **(B)** Combined effect of biochar and gypsum on water use efficiency of stressed and non-stressed plants. **(C)** Effect of gypsum on water use efficiency of stressed and non-stressed plants.

Comparison of WUE of control and biochar treated plants is presented in **Figure 6A** depicting highly significant results (*P* ≤ 0.01). Like other treatments biochar has also improved the WUE of Lady’s finger as compared to control. At 100% F.C. of all treatments (0% B, 1% B, and 3% B) a slight change was observed in WUE weekly throughout the experiment. An increased trend was observed in 1 and 3% B (0.8 and 0.83–1.14 and 1.16 mmolmol^-1^) as compared to control (0% B) where it was just 0.56 mmolmol^-1^. On the other hand at 60% F.C. (0% B, 1% B and 3% B) it was increased throughout the experiment. It has shown maximum value with 1% B (4.6 mmolmol^-1^) as compared to 0 and 3% B where it was (1.6 and 3.8 mmolmol^-1^). Therefore, 1% biochar at 60% F.C. was proved to be more effective in increasing the WUE of Lady’s finger as compared to other treatments.

Biochar along with any fertilizer has also improved the WUE. WUE of control and combination of biochar and gypsum is presented in **Figure 6B**. At 100% F.C. same trend was observed for all the treatments (0% B + 0% G, 1% B + 0.1% G and 3% B + 0.1% G) and maximum value was obtained at with 1% B + 0.1% G. In comparison, at 60% F.C. (0% B + 0% G, 1% B + 0.1% G and 3% B + 0.1% G) among all treatments of biochar and gypsum, WUE was seen to increase throughout the experiment. Similarly, 1% biochar along with gypsum also proved to be more effective.

Comparison of WUE between control and gypsum is presented in **Figure 6C**. At 100% F.C. of all treatments (0% G and 0.1% G) very slight change was observed in all treatments. But in case of 60% F.C., (0% G and 0.1% G) significant increase was observed weekly throughout the experiment. In these treatments (60% F.C. + 0% G and 60% F.C. + 0.1% G), higher WUE was observed in gypsum treated plants (2.21 mmolmol^-1^) as compared to control where it only increased upto 0.91 mmolmol^-1^ at the end of the experiment.

### Biomass

**FIGURE 7:**
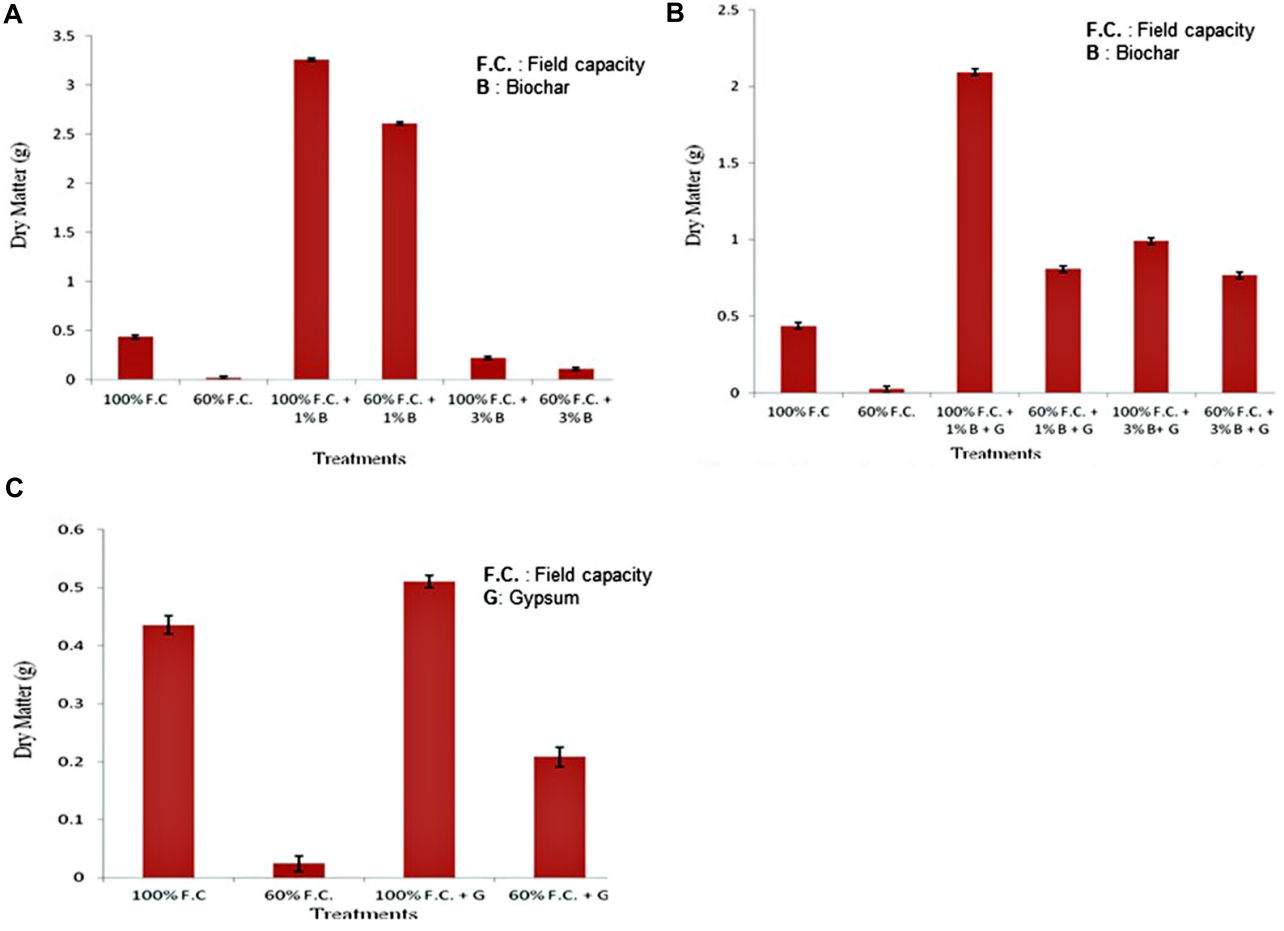
**(A)** Comparison of dry weight of plants between control and biochar treatments under stressed and non-stressed conditions. **(B)** Comparison of dry weight of plants between control and combination of biochar and gypsum treatments under stressed and non-stressed conditions. **(C)** Comparison of dry weight of plants between control and gypsum treatments under stressed and non-stressed conditions.

At the end of experiment, plants were harvested. Fresh and dry weights were calculated for each plant. Biomass of control and biochar treatments is graphically presented in **Figure 7A**. Dry matter was increased in all non-stressed plants (100% F.C. + 0% B, 100% F.C. + 1% B and 100% F.C. + 3% B). Among all these treatments it was higher (3.261 g) in 1% B treatment as compared to other stressed (60% F.C.) and non-stressed plants (100%).

Comparison of control and combination of biochar and gypsum treatment is presented in **Figure 7B**. At 100% F.C. (0% B + 0% G, 1% B + 0.1% G and 3% B + 0.1% G) maximum dry matter was found in 1% B + 0.1% G (2.093 g) than other treatments. On the other hand in stressed plants, i.e., at 60% F.C. (0% B + 0% G, 1% B + 0.1% G, 3% B + 0.1% G) it was decreased due to water stress.

Comparison of control and gypsum treatments is shown in **Figure 7C**. In non-stressed plants an increased biomass was found as compared to stressed plant. At 100% F.C. (0% G and 0.1% G) higher biomass was found in gypsum treated plants (0.511 g) as compared to control (0.436 g).

## Discussion

A better understanding of different strategies is required to achieve drought resistance to cope with the water deficit. WUE is the ability of the crop to produce biomass per unit of water transpired. In this scenario of water stress, WUE can be regarded as an important adaptive trait to drought environment. Previous works have revealed the physiological basis of drought stress in different plants. The present study was made to check the potential of biochar along with gypsum in improving WUE under water stress conditions to study the effect of drought on growth parameters in *A. esculentus* L. Moench (Lady’s Finger).

Soil used for the experiment, represented typical water scares and stressed sandy loam, from dry sub-tropical conditions, with low organic matter, neutral pH, having EC 3.03 μS/cm. It has been observed that biochar and gypsum gave the positive response toward an overall improvement of the studied soil parameters. While pH, EC, moisture content and organic matter was increased in all treated plants as compared to the control soil. Our results followed similar trends established in different studies involving application of biochar in increasing soil pH and EC when applied on tomato (Mustafa et al., 2010) as well as on rice (Zhang et al., 2012). On the other hand it was found that 1% biochar application rate was the more effective treatment compared to other rates. Our findings coincide with a significant increase in plant height of tomatoes with treatment of 1% biochar as compared to 0 and 3% biochar (Ellen et al., 2010). Howard (2011) reported the similar pattern of increase in plant height for corn and soybean by increasing the rate of biochar from 1.5 to 3% while, decreased compared to control level by increasing the rate of biochar upto 6%. We have found the positive effect on plant growth with application of various combinations of biochar and gypsum, e.g., 1% biochar along with 0.1% gypsum as the best combination to increase plant height in control soils at the same time for stressed plants too. Similarly Mustafa et al. (2010) reported that plant height was higher in biochar treatments along with fertilizer as compared to biochar alone, while Sumner and Larrimore (2006) reported similar pattern with gypsum and biochar for cotton crop.

It has been reported that leaf area of plants decreased by decreasing the amount of water. McGiffen et al. (1992) reported 50% reduction in leaf area increase on restricting the water supply of plants. In our study, leaf area was significantly increased up to 3 weeks when the F.C. was maintained but after that increasing rate was less in all stressed plants (60% F.C.) as compared to non-stressed (100% F.C.). Similarly, it has been investigated that 60% maintained F.C. had enough moisture to increase the leaf area as well as the Tr (Masinde et al., 2006). Sankar et al. (2008) reported similar trend of leaf area reduction, with the application of same rate of stress (60%) applied to *A. esculentus* L. Moench. Several other studies report similar results, for other higher plants (Thakur and Kaur, 2001; Reddy et al., 2004).

Agreeing with our findings of a significant reduction in stomatal conductance with stress application, Galmés et al. (2007) reported that different plant species showed decrease in stomatal conductance with water stress. Besides this, a higher stomatal conductance indicated that plants can survive under severe drought conditions for longer period of time (Gindaba et al., 2005). Biochar has been found to increase the stomatal conductance as compared to control (Solaiman et al., 2010). On the other hand, Kusvuran (2012) reported a continuous decline in stomatal conductance in melon (*Cucmis melo*) with no soil amendment. Moreover, same trend was also observed in chickpea (Mafakheri et al., 2010) and *Impatiens capensis* (Heschel and Rignos, 2005). Our study plants responded to drought by decrease in leaf area as well as the transpiration, agreeing with the trend established by Jones (1992).

Moreover, Tr was declined due to reduction in leaf area of stressed plants (Borrell et al., 2000). On the contrary it has also been reported that decline in Tr is due to reduction in stomatal conductance rather than decrease in leaf area (Liu and Stutzel, 2002). Tr was also reduced when the same F.C. (60%) was maintained for Lady’s finger plants by Sankar et al. (2008). Tr of control (no soil amendment) was zero at the end of experiment rather than a bit high in biochar and gypsum treatments. It showed that biochar and gypsum has given the stability to respond under water stressed environment.

Our findings for increase of Pns, with application of Biochar tallied with the findings of biochar increased the Pn of wheat (*Triticum aestivum*) and clover (*Trifolium subterranean*) under drought stress condition as compared to control and fully watered plants (Blackwell et al., 2007; Solaiman et al., 2010). Similar results were found in tobacco plant indicating that photosynthesis and stomatal conductance do not always have direct relationship (Von-Caemmerer et al., 2004). In contrast photosynthesis also decreased in numerous species of *Robinia pseudoaccacia* (Wang et al., 2007) and almond specie (Rouhi et al., 2007). Besides this an increasing trend was observed in Pn of clover (*Trifolium subterranean*) with biochar treated plants under drought stressed conditions (Solaiman et al., 2010). Ippolito et al. (2011) reported that 2% of biochar increased 3–7% of moisture content that enhanced the rate of photosynthesis as well. Water stress in a crucial abiotic factor that considerably influence the plant in many ways. In a study by Azhar et al. (2011) the photosynthetic rate showed non-significant reduction from 100% F.C. to 80% F.C. but increased at 60% F.C. as observed in our case. Such results suggest that there exist certain plants which perform better in water stress environments and thus cultivation of such plants under drought stress conditions should be encouraged.

Coincided with other studies for increased WUE in *A. esculentus* L. Moench, some other plant species also reported to show improved WUE under drought stress conditions as compared to fully watered plants (Yin et al., 2005; Liang et al., 2006). Many studies have shown same results that decrease in Tr and increase in Pn have known to increased WUE (Chen et al., 2000). Similarly, Kimball et al. (1994) reported that Pn was increased due to increased CO_2_ concentration and finally WUE was also increased due to increased net photosynthesis. In contrast, Murray (1995) reported that WUE was increased due to decreased stomatal conductance and Rogers et al. (1994) reported that CO_2_ was increased due to Tr and so increased WUE was due to decreased Tr.

In the present study, Tr was reduced and Pn was increased that caused an increase in WUE. In previous studies WUE was also determined for different species such as sweet potato (4.89 mmolmol^-1^), rice (3.28 mmolmol^-1^), soybean (3.68 mmolmol^-1^) and maize (9.23 mmolmol^-1^). In the same study WUE was improved due to increased photosynthesis and decreased transpiration (Jianlin et al., 2008).

In case of our findings for increase in biomass, a similar 20% increase in dry weight was obtained in biochar along with gypsum applied plants as compared to biochar and gypsum alone (Mustafa et al., 2010). For our findings of change in dry weight for the gypsum treatment of stressed and control, a comparable finding (Jones, 1992) for dry matter reduction in stressed plants as compared to non-stressed plants due to low rate of transpiration and small leaf area. Similar results were also found when the same rate of water stress was imposed on the plants of Lady’s finger (Sankar et al., 2008). Several studies have shown a higher photosynthetic rate under the optimal water (60% level of F.C.) conditions (Azhar et al., 2011). In general, there is a positive relationship between photosynthesis and biomass accumulation of plants, particularly with the addition of biochar (Reddy and Das, 1986; Peng et al., 1991; Hossain et al., 2012). The results of our study showed a higher rate of photosynthesis at 60% F.C., but the highest biomass was recorded at water level of 100% F.C. This means that other physiological processes such as the high rate of respiration might have hindered the biomass accumulation of plant. However, more research is needed to establish the relationship between the rate of respiration, photosynthesis and biomass accumulation at different soil amendments and water stress levels.

Different plants can withstand certain level of pH and lady’s finger can suitably grow under 6–8 pH. With the amendment of biochar, it overcomes the negative effects of pH at 1%. But at 3% concentration, the pH increased which resulted in reduction of positive effect of biochar (Howard, 2011). Our experiment depicted the similar situation of good results with 1% biochar as compared to 3%.

## Conclusion

Water being essential in growth and development of plants, a specific amount of water is needed for optimum growth. WUE in rain fed areas is the most important limiting factor for the plant growth. Increasing temperature of earth due to anthropogenic activities, Climate changes and severe weather conditions are reasons for flash floods, drought and glacial retreat, destabilized watersheds are heading toward water scarcity. Pakistan is facing increasing drought stress, being an agriculture based economy. In the current scenario soil amendments can prove to be the best methods to overcome drought stress.

In this study biochar and gypsum were used to improve the infiltration rate and water holding capacity of soil in which *A. esculentus* L. Moench seedlings were grown for 4 weeks and then stress treatments were imposed. The variations were noted and assessment of growth as well as physiological parameters was done in experimental conditions.

Among all treatments, 1% biochar alone as well as along with gypsum gave significant results for all parameters as compared to control and other treatments. It is finally concluded that biochar alone is a better strategy to promote plant growth specifically of *A. esculentus* L. Moench, compared to the application of gypsum and combination of biochar and gypsum under both stressed and non-stressed conditions. Furthermore, at 60% F.C. Lady’s finger showed resistance as well as improved WUE. Therefore, it is considered beneficial in water stressed regions.

## Conflict of Interest Statement

The authors declare that the research was conducted in the absence of any commercial or financial relationships that could be construed as a potential conflict of interest.
